# Associations between skating mechanical capabilities and off‐ice physical abilities of highly trained teenage ice hockey players

**DOI:** 10.1002/ejsc.12184

**Published:** 2024-09-10

**Authors:** Julien Glaude‐Roy, Julien Ducas, Jean‐François Brunelle, Jean Lemoyne

**Affiliations:** ^1^ Service de l'activité physique et sportive Université du Québec à Trois‐Rivières Trois‐Rivières Québec Canada; ^2^ Department of Human Kinetics Université du Québec à Trois‐Rivières Trois‐Rivières Québec Canada; ^3^ Laboratoire de recherche sur le hockey Université du Québec à Trois‐Rivières Trois‐Rivières Québec Canada

**Keywords:** agility, force‐velocity profile, power, speed, sprinting

## Abstract

This study examines the associations between force and velocity characteristics of forward skating and off‐ice speed, agility, and power of highly trained teenage ice hockey players. Players attending the Quebec ice hockey federation's off‐season evaluation camp were invited to participate in this study. Final sample consists of 107 highly trained teenage ice hockey players (Males: *n* = 38; 13.83 ± 0.38 years; Females: *n* = 69: 14.75 ± 0.90 years). Individual force–velocity profiles (F–V) were determined during a 44 m skating sprint. Off‐ice speed, agility, and power were measured using 30 m sprint, 5‐10‐5 agility, and standing long jump. Associations between F–V mechanical capabilities and off‐ice indicators were analyzed with correlational analyses and multivariate analysis of covariance (MANCOVA). Results of pooled data indicate that the three off‐ice measures had moderate associations with F_0_ and V_0_ and large associations with P_max_. Associations with Rf_max_, D_rf,_ and S_fv_ were moderate to small. F_0_ had stronger associations with off‐ice performance in female players while V_0_ was more important with male players. MANCOVA identified 5‐10‐5 times as the better predictor for F_0_ while 30 m sprints times better predicted V_0._ To maximize physical attributes of skating ability, practitioners are encouraged to focus on a general physical preparation for highly trained teenage players. Prioritizing types of exercises that use change of direction or acceleration and linear speed should have distinct effects on F_0_ and V_0_ on the ice.

## Highlights

- A good general physical preparation is important for highly trained teenage ice hockey players as maximal running speed, change of direction ability, and leg power in the horizontal plane are largely associated with mechanical capabilities during maximal skating performance.
- Change of direction ability tends to be associated with the ability to generate force at low velocities on the ice (F_0_) while maximal running speed tends to be associated with the ability to generate force at high velocities on the ice (V_0_).
- Off‐ice abilities are more strongly associated to the ability to generate force at low velocities on the ice (F_0_) in highly trained female players than in highly trained male players.

## 1 INTRODUCTION

Ice hockey is a team sport with intermittent bouts of high‐intensity efforts interspersed with relatively short periods of recovery (Douglas & Kennedy, 2020). On average, players perform approximately 18 high‐intensity activities per period including forward and backward sprints counting for roughly 20% of effective playing time (Brocherie et al., 2018). During off‐season, strength and conditioning coaches devote substantial efforts to integrate approaches designed to improve acceleration, power, and speed. With young ice hockey players, doing so appears to be associated with skating speed in the upcoming season (Haukali & Tjelta, 2016). Despite the multiple approaches (traditional training, contrast approaches, plyometrics, etc.) designed to improve players' functional performance, for a single training method or a specific physical quality, each individual's adaptation process is unique (Donskov, 2016). In line with this assumption, Jiménez‐Reyes et al. (2017) recently proposed an original methodology for individualized strength and power training programs using athlete's force–velocity profile (F–V). According to these authors, F–V offers a practical and effective way to individualize training programs based on athletes' individual profiles by reinforcing qualities identified as “deficient” during the assessment.

In its initial stage of development, the sprinting F–V was validated using specialized treadmills and tracks with embedded force plates demonstrating that velocity of the center of mass as a function of time follows a mono‐exponential function during maximal sprints (Morin et al., 2012; Rabita et al., 2015). Subsequently, simple practical field methods using split times or instant velocity were validated to accommodate practitioners in their purpose to optimize training programs (Samozino et al., 2016). F–V variables include maximal theoretical force relative to body mass (F_0_), maximal theoretical velocity (V_0_), maximal theoretical power relative to body mass (P_max_), force velocity slope (S_fv_), the ratio of the horizontal component of the resultant ground reaction force (RF_max_), and rate of decrease of the ratio of the horizontal component of the ground‐reaction force (D_RF_) (see Table 1 for detailed definitions of mechanical variables and Perez et al. (2019) for detailed equations). Computed variables are then used to determine if athletes are more “force deficient” or “velocity deficient”.

**TABLE 1:** Definitions of force–velocity mechanical variables.

Variable	Definition	Units
F_0_	Maximal theoretical horizontal force relative to body mass	N*kg^−1^
V_0_	Maximal theoretical horizontal velocity	m*s^−1^
P_max_	Maximal theoretical horizontal power relative to body mass	W*kg^−1^
D_rf_	Rate of decrease of the ratio of the horizontal component of the ground‐reaction force to the corresponding resultant force	%
Rf_max_	Maximal ratio of the horizontal component of the ground‐reaction force to the corresponding resultant force	%
S_FV_	Slope of the force–velocity relationship applied to the center of mass	N*m*s^−1^

Alternative methods such as 3D kinematics, 3D accelerometry or insole dynamics were also used to analyze ice hockey acceleration and skating speed and identify players' strengths and deficiencies (Buckeridge et al., 2015; Budarick et al., 2020; Mazurek et al., 2023). These methods allowed for reliable and precise biomechanical analysis on the ice of forward skating. For example, collection of data allowed for the observation of differences in joint angles and muscle activation from a “running” phase corresponding to the first three steps and “gliding” phase at the sixth step (Budarick et al., 2020). Yet, they generally require expensive equipment or long set up times which may not be adequate in applied settings. In comparison, the approach developed by Samozino et al. (2016) has been shown to be reliable on the ice (Perez et al., 2019) and allows for the assessment of force and velocity characteristics on the ice. Considering the importance of strength and speed in ice hockey, this avenue can potentially lead to interesting developments to enhance skating performance.

In 2016, Morin and Samozino published guidelines to help practitioners with the interpretation of F–V mechanical variables. They suggested that in relation with athletes' sprinting performance, short accelerations (<20 m) are associated with higher values of F_0_ and Rf_max_ while longer distances are associated to V_0_ and D_rf_ (>30 m). Recent research with other team sports are congruent with the proposed model. For example, Baena‐Raya et al. (2020) explored associations with change of direction ability demonstrating the stronger associations between 505 change of direction performance and F_0_. In a study with rugby athletes, assisted and resisted sprint training had opposite effects on the F–V with the magnitude of effects influenced by the initial F–V and assisted sprints correlating with V_0_ (Lahti et al., 2020).

Research oriented toward ice hockey players' F–V using Samozino et al.’s (2016) methodology is fairly scarce with, to our knowledge, very few published articles (e.g., <10). A majority of studies including this method established the reliability of various field methods including radars (Perez et al., 2019), multiple loaded methods (Perez et al., 2022), and a combination of split times and high‐speed video cameras (Stenroth et al., 2020). Perez et al. (2019) led the work specific to ice hockey demonstrating that skating velocity follows the same mono‐exponential function as observed during running. Later, Perez et al. (2021) demonstrated the limited associations between skating and off‐ice F–V which give distinct information on a player's mechanical abilities. These results provide limited guidelines to practitioners eager to prescribe and individualize training during off‐season in order to maximize muscular components of skating performance. In fact, a better understanding of the associations from a more global or general perspective (e.g., off‐ice) to sport‐specific actions (e.g., on‐ice sprints) is crucial in terms of optimizing performance in competitive ice hockey (Novák et al., 2019; Secomb et al., 2021).

When considering off‐ice protocols or exercises possibly associated with on‐ice performance, standing long jumps (SLJ), vertical jumps, ≈36 m sprints, change of direction ability, and maximal squats are the most frequently cited examples (Delisle‐Houde et al., 2019; Henriksson et al., 2016; Janot et al., 2015; Novák et al., 2019). As mentioned, the associations between these tests and mechanical properties of forward skating have not been explored. Thus, this study aims to explore the associations between the force and velocity characteristics of skating and off‐ice speed, agility, and leg power of highly trained teenage ice hockey players. Previous observations in sprinting demonstrated that female athletes tend to have more “force‐oriented” profile than males (Devismes et al., 2021; Haugen et al., 2020). Hence, to take a deeper look in the associations between off‐ice performance and skating force and velocity characteristics a second aim of this study is to explore the influence of biological sex in observed associations. Identifying relationships between these variables should help practitioners target specific exercises in training and maximize desired changes to individual players' skating F–V.

## 2 MATERIALS AND METHODS

### 2.1 Context and participants

Each year, the province of Quebec’ ice hockey federation (Canada) chooses the province's most promising male and female hockey players through a rigorous evaluation process based on their performance during regular season. These players are invited to an off‐season (summer) development camp, where the best players are selected to compete at the national level. Despite the potential impact of physical deconditioning that tends to occur among competitive hockey players (Chiarlitti et al., 2021; Delisle‐Houde et al., 2019), testing these players off‐season was the most appropriate scenario for assessing athletes' abilities. Data were collected from a full testing protocol designed to identify players part of the selection process for males under 15 years old (U15) and females under 17 years old (U17). These players were classified as highly trained teenage athletes because of their high training loads and competition at the national level (McKay et al., 2021). A total of 199 players (86 males, 113 females) were invited to both camps. Goaltenders (*n* = 18; 50% boys) were excluded from the study because of the unique nature of their position (Marcotte‐L’Heureux et al., 2021). Injured players were also excluded from analysis given they could not complete all the sprint, agility or jumping tests. Incomplete datasets were also excluded from analysis (technical problems occurred with radar testing during data collection), which resulted in a final sample of 107 participants (38 males 13.83 ± 0.38 years, 69 females 14.75 ± 0.90 years). The project was approved by the ethics board of the researchers' institution (CER‐21‐278‐07.29). Article 21 of the civil code of the province of Quebec stipulates: “*Consent to research that could interfere with the integrity of a minor may be given by the person having parental authority or the tutor*. *A minor 14 years of age or over*, *however*, *may give consent alone if*, *in the opinion of the competent research ethics committee*, *the research involves only minimal risk and the circumstances justify it*.” Therefore, parents and players were informed of the research project and only written consent from the players was obtained. Written consent of parents for 11 players who were under 14 years of age was obtained.

### 2.2 Study design

In both development camps, the protocol consisted of off‐ice fitness tests (*n* = 6) and on‐ice tests (*n* = 2). The full testing protocol, which included tests measuring speed, agility, leg power, upper‐body strength and power, cardiovascular endurance, mobility, and anthropometric values (detailed description is available in: Lemoyne et al., 2022). Tests performed included a 30‐m sprint test, the 5‐10‐5 shuttle run, counter‐movement jump, SLJ, grip strength, the 20 m shuttle run, and the Y‐balance test. Sub‐groups of 24 players were given 60 min to complete the tests in random order with the exception of the 20 m shuttle run, which had to be completed all at once in an additional 30‐min period after the other tests. On‐ice tests measured linear speed and agility. The 45‐min session, started with an 8‐min general warmup, followed by short skating accelerations. Then player completed the 44.8 m maximal skating test first followed by an agility test. Assessments were conducted on two separate days (day‐1 = on‐ice; day‐2 = off‐ice). Only data for the skating F–V and 30 m sprint, 5‐10‐5 shuttle run, and SLJ are provided in this article. Players' characteristics (anthropometric measures, age, and biological sex) are presented in Table 2.

**TABLE 2:** Male and female descriptive statistics for skating force–velocity mechanical variables and performance indicators.

	Females				Males			
	Minimum	Maximum	Mean	SD	Minimum	Maximum	Mean	SD
Mass (kg)	45.00	86.50	62.02	8.74	46.60	80.70	63.08	7.97
Stature (cm)	145.00	178.00	164.84	5.89	157.50	185.00	171.23	7.10
F_0_ (N/kg)	3.01	5.00	3.79	0.35	3.87	5.73	4.65	0.41
V_0_ (m/s)	8.00	9.92	9.08	0.38	8.56	10.87	9.83	0.46
P_max_ (W/kg)	6.98	11.52	8.60	0.90	8.60	13.87	11.43	1.12
Rf_max_ (%)	18.93	36.76	27.62	3.06	25.70	39.46	33.40	3.29
D_rf_ (%)	−5.18	−3.22	−4.09	0.39	−5.61	−3.83	−4.56	0.43
S_fv_ (%)	−54.27	−32.44	−41.89	4.14	−59.25	−39.65	−47.48	4.79
30 m sprint (s)	4.68	5.60	5.13	0.20	4.45	5.26	4.79	0.21
5‐10‐5 (s)	5.10	6.26	5.73	0.23	5.02	5.75	5.33	0.16
SLJ (cm)	160.00	224.00	193.93	15.81	194.00	258.00	226.45	14.72

Abbreviations: SD, standard deviation; SLJ, Standing long jump.

### 2.3 Skating force–velocity profile

Skating performance was assessed over 44.8 m to allow players to reach maximal velocity (Stastny et al., 2023). To determine the skating F–V of each player, instant velocity was collected following the guidelines of Perez, Guilhem, and Brocherie (Perez et al., 2019). The method shows “acceptable” inter‐trial and test–retest reliability (intra‐class correlation coefficients (ICC) ≥0.75 and coefficient of variation [CV] ≤ 10%) (Perez et al., 2019). Before each sprint, players were instructed to render maximal effort over the entire distance. They started in a crouched position while waiting for the evaluator's signal to start. Each player had two trials to register their fastest sprint. Instant velocity was collected at 46.875 Hz using a Stalker Pro Radar II (Stalker Sport). The radar was placed 3 m behind the starting line and 1 m above ground approximately at the height of players' center of mass. Mechanical capabilities were computed using a custom R script. For each trial, raw data were imported in R studio. Then, data prior to the start and after the maximal velocity plateau were discarded. Remaining data were fitted with a mono‐exponential function and mechanical variables were calculated (Perez et al., 2019; Samozino et al., 2016). To improve reliability of F–V variables, the average value of both trials was used for analysis (Edwards et al., 2022; Perez et al., 2019; Simperingham et al., 2019).

### 2.4 Off‐ice speed, agility and leg power

Sprints, change of direction ability, and standing long jumps were tests chosen for the talent identification process as that are commonly associated with skating performance (Delisle‐Houde et al., 2019; Henriksson et al., 2016; Janot et al., 2015; Novák et al., 2019). Off‐ice linear speed was assessed with a 30‐m sprint. Times were collected using Swift Speedlight photocells single beam laser timing gates placed on the start and finish lines (Swift Performance). Players adopted a standing stance inside the first gate and were instructed to give a maximal effort. The “steady” feature used was used to trigger the timing gates. This feature starts the timer when the player exits the laser beam. Players had two trials with 3 minutes rest between trials. Only the fastest sprint was used for analysis.

To assess off‐ice agility, players ran a 5‐10‐5 shuttle utilizing the same timing gate system employed for the 30‐m sprints. Three lines were positioned 5 m apart. Players initiated the run from the center line with one hand touching the ground. The run times were captured through a singular Swift timing gate positioned above the center line, incorporating the “steady” feature to initiate the timing gate. Upon instruction from the evaluator, participants 1‐ ran to one side, 2‐ touched the line with their hand, 3‐ ran to the opposite side, 4‐ touched the line with their hand, and 5‐ ran back passed the center line as fast as possible. Each player had a single attempt on both sides, with only the fastest time retained for analysis (Lam et al., 2018).

Leg power in the horizontal plane was assessed with three SLJs. Players stood in a static position with both feet behind the take‐off line. Using a countermovement and double‐arm swing they had to jump as far as possible landing without moving their feet or losing balance. Performance was the distance between the take‐off line and closest heel. The longest jump was used for analysis.

### 2.5 Statistical analyses

Players' characteristics (age, mass, and stature) and variables from skating F–V (F_0_, V_0_, P_max_, RF_max_, D_RF,_ and S_fv_) and off‐ice tests were processed using SPSS Statistics 28.0. Normality of distribution for each variable was confirmed by verifying skewness and kurtosis (values < 1 and < 2 respectively). Preliminary analyses revealed no violation of normality for each distribution. Descriptive statistics were computed to verify baseline measures. Reliability of F–V mechanical capabilities with adolescent ice hockey players was confirmed by computing ICC and coefficients of variations (CV) for both trials. All variables had ICC values ≥ 0.90 and CV ≤ 10%. Partial Pearson's correlation coefficients were calculated to explore the relations between players' skating F–V and off‐ice performance while controlling for biological sex in order to account for differences between male and female players. Pearson's correlation coefficients were computed for male and female players in order to compare the differences in sexes. In order to verify the contribution of each off‐ice tests as predictors of skating force and velocity characteristics, multivariate analysis of variance (MANCOVA) was conducted. F_0_ and V_0_ were introduced simultaneously as dependent variables, as they represent estimations of different parts of maximal skating ability (e.g., acceleration and maximal velocity). The three off‐ice tests (e.g., SLJ, 30 m sprint time, and 5‐10‐5 time) were set as covariables (Aliberti et al., 2023). Preliminary analyses demonstrated equal covariances matrices (Box's *M* = 3.960 and *p* = 0.276) and equality of error variances for both dependent variables (Levene's test: F_F0 (1,105)_ = 3.184 and *p* = 0.077; F_V0 (1,105)_ = 1.066 and *p* = 0.304). As recommended by Hopkins et al. (2009), the magnitude of effect sizes was considered trivial (*r* < 0.10), small (*r* = 0.10–0.29), moderate (*r* = 0.30–0.49), large (*r* = 0.50–0.69), very large (*r* = 0.70–0.89), nearly perfect (*r* = 0.90–0.99), and perfect (*r* = 1.00).

## 3 RESULTS

**FIGURE 1:**
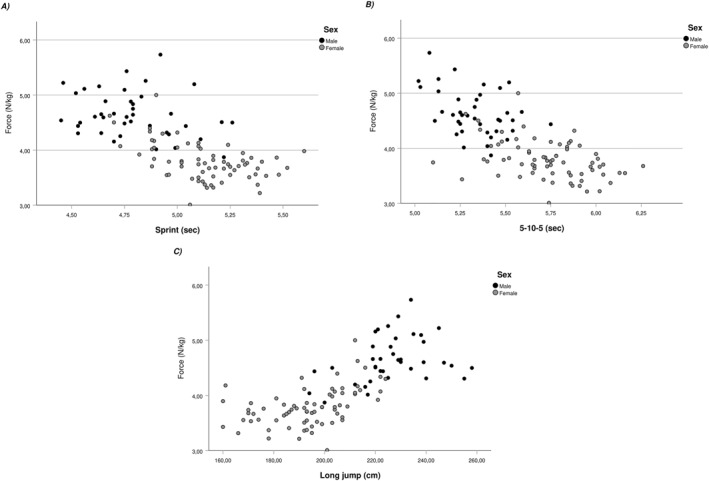
Associations between force and off‐ice abilities. (A) Association between force and sprint time *r*_pooled_ = −0.39, *r*_male_ = −0.25, and *r*_female_ = −0.50. (B) Association between force and 5‐10‐5 time *r*_pooled_ = −0.39, *r*_male_ = −0.41, and *r*_female_ = −0.40. (C) Association between force and standing long jump performance *r*_pooled_ = 0.42, *r*_male_ = 0.33, and *r*_female_ = 0.46.

**FIGURE 2:**
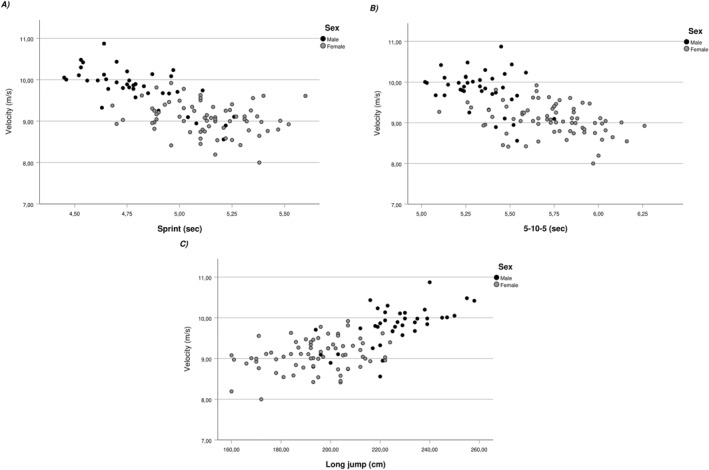
Associations between velocity and off‐ice abilities. (A) Association between velocity and sprint time *r*_pooled_ = −0.43, *r*_male_ = −0.71, and *r*_female_ = 0.22. (B) Association between velocity and 5‐10‐5 time *r*_pooled_ = −0.39, *r*_male_ = −0.32, and *r*_female_ = −0.32. (C) Association between velocity and standing long jump performance *r*_pooled_ = 0.39, *r*_male_ = 0.59, and *r*_female_ = 0.27.

Descriptive statistics for male and female players are presented in Table 2. Associations between off‐ice variables and F_0_ for pooled, female, and male data are presented in Figures 1 and 2 for V_0_. Pooled 30 m sprint times were moderately associated with F_0_ (*r* = −0.39, *p* < 0.001) and V_0_ (*r* = −0.43, *p* < 0.001), largely associated with P_max_ (−0.53, *p* < 0.001), and had a small association with Rf_max_ (*r* = −0.25, *p* = 0.01). The 5‐10‐5 times were moderately associated with F_0_ (*r* = −0.39, *p* < 0.001), V_0_ (*r* = −0.33, *p* < 0.001), and *P*_max_ (−0.49, *p* ˂ 0.001) and had small, associations with S_fv_ (*r* = 0.20, *p* = 0.038) and Rf_max_ (*r* = −0.24, *p* = 0.014). SLJ was moderately associated with F_0_ (*r* = 0.42, *p* < 0.001) and V_0_ (*r* = 0.39, *p* < 0.001), largely associated with *P*_max_ (*r* = 0.54, *p* < 0.001), and had small associations with S_fv_ (*r* = −0.21, *p* = 0.035) and Rf_max_ (*r* = 0.28, *p* = 0.004). None of the off‐ice variables were significantly associated with D_rf_.

Females' 30 m sprint time was largely associated with F_0_ (*r* = −0.50, *p* < 0.001) and *P*_max_ (*r* = −0.52, *p* < 0.001) and moderately associated with S_fv_ (*r* = 0.37, *p* = 0.002), Rf_max_ (*r* = −0.30, *p* = 0.011) and D_rf_ (*r* = 0.35, *p* = 0.003). 5‐10‐5 time was moderately associated with F_0_ (*r* = −0.40, *p* < 0.001), V_0_ (*r* = −0.32, *p* = 0.007), and *P*_max_ (*r* = −0.48, *p* < 0.001) and had a small association with S_fv_ (*r* = 0.24, *p* = 0.044). SLJ was moderately associated with F_0_ (*r* = 0.46, *p* < 0.001), S_fv_ (*r* = −0.31, *p* 0.008), Rf_max_ (*r* = 0.30, *p* = 0.012), and D_rf_ (*r* = −0.30, *p* = 0.012), largely associated with *P*_max_ (*r* = 0.52, *p* < 0.001) and had a small association with V_0_ (*r* = 0.27, *p* = 0.022). Males' 30 m sprint time was very largely associated with V_0_ (*r* = −71, *p* < 0.001) and largely associated with *P*_max_ (*r* = −0.55, *p* < 0.001). 5‐10‐5 time was moderately associated with F_0_ (*r* = −0.41, *p* = 0.010), V_0_ (*r* = −0.32, *p* = 0.044), and Rf_max_ (*r* = −0.32, *p* = 0.050) and was largely associated with *P*_max_ (*r* = −0.53, *p* < 0.001). SLJ was moderately associated with F_0_ (*r* = 0.33, *p* = 0.041) and was largely associated with V_0_ (*r* = 0.59, *p* < 0.001) and *P*_max_ (*r* = 0.57, *p* < 0.001).

In order to verify the contribution of each off‐ice tests as predictors of skating force and velocity characteristics, MANCOVA was conducted simultaneously on F_0_ and V_0_. For F_0_, a significant effect was observed: F (3, 103) = 49.635, *p* < 0.001, *η* ^2^ = 0.59. Performances in the long jump (*β* = 0.010, *p* < 0.001, *η* ^2^ = 0.11) and the change of direction tests (*β* = −0.600, *p* = 0.002, *η* ^2^ = 0.09) were significantly associated with F_0_. For V_0_, a significant effect was also observed: *F* (3, 103) = 36.848, *p* < 0.001, *η* ^2^ = 0.52. Performances in the long jump (*β* = 0.008, *p* = 0.007, *η* ^2^ = 0.07) and in the 30 m sprint (*β* = −0.558, *p* = 0.025, *η* ^2^ = 0.05) were significantly associated with V_0_.

## 4 DISCUSSION

Acceleration and speed on the ice are crucial factors that determine the overall skating performance of teenage ice hockey players which can be optimized during off‐season conditioning (Haukali & Tjelta, 2016). Recent developments in the analysis of sprinting capabilities using F–V profiling suggest distinct adaptations according to chosen training modalities (Lahti et al., 2020). In the unique context of ice hockey, associations between off‐ice abilities and skating mechanical capabilities is still unknown. Perez et al. (2021) have explored agreement across different F–V methods (e.g., skating, sprinting, and jumping) recommending to choose skating F–V for individualized training prescription. To our knowledge, this study is the first to explore associations between force and velocity characteristics of skating and off‐ice speed, power, and change of the direction ability of highly trained teenage ice hockey players. When examining pooled data in Figures 1 and 2, moderate to large associations were present between the mechanical properties (F_0_ and V_0_) and all off‐ice abilities. Previous work had identified sprints, agility, and SLJ as exercises associated with skating performance (Delisle‐Houde et al., 2019; Henriksson et al., 2016; Janot et al., 2015; Novák et al., 2019). The present results are in line with these observations and emphasize the importance of general physical preparation for young competitive ice hockey players.

Results refine previous observations giving added information on how improvements off the ice may be associated with performance on the ice. Even though all off‐ice measures used in this study contribute to force and velocity characteristics in skating, MANCOVA suggests distinct associations. SLJ performance and change of direction ability may translate more to F_0_ while the 30 m sprint performance may translate to V_0_. Morin and Samozino (2016) have previously stated that sprints over short distances (<20 m) are associated to F_0_ while sprints over longer distances (>20 m) be associated with V_0_. In a different study, Slavicek et al. (2022) have observed associations between off‐ice change of direction and on‐ice agility. Combined to the present results, the exercises performed during off‐season training may translate to specific adaptations to skating performance at the start of the season or during early season selection camps. Working on improving leg power and change of direction ability could lead to better improvements in skating acceleration and skating agility (as observed by Slavicek et al., 2022) while improving sprinting performance could lead to improved maximal skating speed.

The second aim of this study, was to explore the influence of biological sex in observed associations between force and velocity characteristics of skating and off‐ice performance. Comparing strength of associations for both groups individually in Figures 1 and 2 exposes these differences. In general, female players' off‐ice performance showed large to moderate associations with F_0_ and moderate to small associations with V_0_. In the contrary, male players' off‐ice performance was moderate to small with F_0_ but very large to moderate with V_0_. Previously, Perez et al. (2021) explored relationships between F–V profiles in skating, running, and vertical jumping tasks and identified F_0_ and P_max_ as strong predictors of skating performance for female players from the French national team. Even though the present results emanate from an entirely different context, the same variables (e.g., F_0_ and P_max_) exhibited the strongest associations with off ice‐performance in highly trained female players. Interestingly, the associations were the opposite with male players for whom V_0_ was the main parameter associated with off‐ice performance. Previous work comparing the F–V capabilities of female and male teenage soccer players reported more “force‐oriented” profiles for female players (Devismes et al., 2021; Haugen et al., 2020). The proposed explanation for these observations was based on the differences in distances traveled during match play where male players tend to sprint over longer distances than females. Gamble et al. (2022) have compared external load of varsity male and female players. They observed that male players skated roughly twice the distance above 24 km/h than female players. Higher training loads at these high speeds could lead male players to have an enhanced ability to generate force at high velocities (V_0_) explaining in part the important associations observed in this study. Another explanation may lie in skeletal muscle fiber type differences between sexes. A meta‐analysis by Nuzzo (2023) showed that males exhibited greater area percentage of all Type II muscle fibers which are associated with the ability to generate speed and power. Both explanations support the observed differences between male and female players from this study.

The design chosen for this study suggest interesting insights on associations between off‐ice performance and mechanical capabilities during maximal forward skating, but certain limitations subsist. First, qualities where assessed in an off‐season setting. Hence, athletes have more time to invest in their physical preparation process, which might lead to the attainment of higher scores in comparison with what would have been observed in season. In other words, participants might have performed better in off‐season settings, diminishing the potential physical deconditioning (Chiarlitti et al., 2021) that occur during a competitive season. Taking a deeper look on how players perform in both contexts is necessary. Secondly, this study presents results for a very specific group of highly trained teenage ice hockey players. Because of their chronical age, it is possible that players, especially males, were at different maturational stages. Previous work by Fernández‐Galván et al. (2022) demonstrated important changes in mechanical capabilities from pre to post peak height velocity for young soccer players which influenced training prescription. Considering maturational stage in ice hockey would certainly help to optimize player development. Thirdly, participants being part of a larger selection process only allowed for the exploration of associations of three off‐ice tasks. Secomb et al. (2021) demonstrated the importance of joint‐angle‐specific hip strength for skating acceleration and change of direction. Including leg strength in off‐ice performance indicators would certainly influence results of MANCOVA and give further insights of associations with skating mechanical determinants. Fourthly, Samozino et al.’s method (2016) is based off radar measurements of speed which don't account for the specific nature of skating (e.g., ice friction and lateral pushing mechanics) or allow for precise detection of strides during skating. Validation of the method with 3D kinematics or insole dynamics would be advised. Finally, conclusions on associations of specific off‐ice indicators with F_0_ and V_0_ should be considered with care because of the observational nature of this study. To our knowledge, effects of any off‐ice intervention aimed at modifying or enhancing skating F–V have yet to be examined. Previous research in rugby demonstrated the importance of exercise selection for specific F–V adaptations (Lahti et al., 2020) and might serve as general guidelines for specific ice hockey interventions. The specific associations observed in this study lead us to believe that interventions comparing different training methods should also lead to specific adaptations on the ice, but remain to be explored.

## 5 CONCLUSION

This study explored associations between force and velocity characteristics of skating and off‐ice speed, agility, and leg power of highly trained adolescent ice‐hockey players. Main results demonstrated strong correlations between all mechanical variables of the skating F–V and off‐ice tasks identified in this study. Secondary results showed distinct associations for F_0_ and V_0_. Both mechanical variables were associated with leg power but change of direction ability related more strongly to F_0_ and speed related more strongly to V_0_. In light of these results, practitioners are encouraged to focus on a good general physical preparation to maximize acceleration and maximal speed on the ice with highly trained teenage players. Even though further research on the topic is necessary, they should also be aware that choices made in the weight room possibly have distinct effects on the skating performance. Focusing training time to change of direction ability or acceleration and short sprinting drills should lead to improved acceleration on the ice while improving linear sprinting speed should help improve maximal skating speed.

## CONFLICT OF INTEREST STATEMENT

Authors declare no conflicts of interest with this research. This research was conducted in the absence of any commercial or financial relationships that could be construed as potential conflicts of interest.

## Data Availability

Data that support the findings of this study are not publicly available. Data are available upon request from the corresponding author JGR.
